# ADAMTSL4, a Secreted Glycoprotein, Is a Novel Immune-Related Biomarker for Primary Glioblastoma Multiforme

**DOI:** 10.1155/2019/1802620

**Published:** 2019-01-08

**Authors:** Zheng Zhao, Ke-Nan Zhang, Rui-Chao Chai, Kuan-Yu Wang, Ruo-Yu Huang, Guan-Zhang Li, Yong-Zhi Wang, Jing Chen, Tao Jiang

**Affiliations:** ^1^Department of Molecular Neuropathology, Beijing Neurosurgical Institute, Beijing, China; ^2^Chinese Glioma Genome Atlas (CGGA), China; ^3^Department of Neurosurgery, Beijing Tiantan Hospital, Capital Medical University, Beijing, China

## Abstract

**Background:**

Researches on immunotherapy of glioblastoma multiforme (GBM, WHO grade IV) have increased exponentially in recent years. As a targeted therapy, a series of biomarkers have been identified in local tumor tissue, while circulating marker which could be detected in the body fluids is still lacking. ADAMTSL4, a secreted glycoprotein, was earlier found to play a critical role in a prognostic signature for primary GBM (pGBM). We aimed to investigate the role of ADAMTSL4 at transcriptome level and its relationship with clinical practice in pGBM.

**Methods:**

A cohort of 88 pGBM patients with RNA-seq data from the Chinese Glioma Genome Atlas (CGGA) was analyzed, and 168 pGBM patients from TCGA were included as validation. Several bioinformatic methods and predictive tools were applied to investigate the ADAMTSL4-associated immune microenvironment status.

**Results:**

We found that ADAMTSL4 was enriched in GBM (WHO grade IV), especially for those with IDH1/2 wild-type and MGMT unmethylated groups. According to the TCGA classification scheme, ADAMTSL4 can act as a potential marker for subtypes with poorer prognosis. Bioinformatic analyses revealed that ADAMTSL4 was significantly correlated to the immune-related processes in GBM (WHO grade IV), especially representing the infiltration of immune cells and complicated tumor microenvironment. Clinically, high expression of ADAMTSL4 was an independent indicator for poor prognosis.

**Conclusion:**

The expression of ADAMTSL4 is closely related to the clinicopathologic characteristics of pGBM. Meanwhile, it may play a critical role in immune-related processes. As a secreted glycoprotein, ADAMTSL4 is a promising circulating biomarker for pGBM, deserving further investigations.

## 1. Introduction

Primary glioblastoma multiforme (pGBM, WHO grade IV) is the most common and fatal neoplasm in the central nervous system [1, 2]. Although comprehensive treatment has been well performed [3, 4], including possible total resection, postoperative radiotherapy, and adjuvant chemotherapy for the past few years, the prognosis of GBM patients remains poor. The median survival is 14.4 months, and a 5-year survival rate is less than 5% [1, 2, 5]. Nonetheless, with the gradually understanding of oncoimmunology in glioma, the fast-growing immunotherapy is shedding a light to the dim prognosis of GBM [6–9].

In general, GBMs with poorer prognosis usually induce more intensive immunoreactions and more complicated tumor microenvironment [6, 10]. For the immune escape mechanism of tumor is overly complex, immune checkpoints have been investigated to be biomarkers for the immune escape of GBM (WHO grade IV) [11, 12], while, so far, molecular markers indicating the status of the immune checkpoint pathways could only be detected in local tumor tissue. New circulating biomarkers should be further detected [13].

ADAMTS-like proteins are secreted glycoproteins, included in the ADAMTS (a disintegrin-like and metalloproteinase domain with thrombospondin type 1 motifs) superfamily, which lacks the prometalloprotease and the disintegrin-like domain typical of this family but contains other ADAMTS domains in precise order [14, 15]. The most widely studied members of ADAMTSL family are ADAMTSL1 and ADAMTSL4. Nowadays, ADAMTSL1 was gradually uncovered the close relationship with breast cancer and chondrosarcoma [16, 17]. As a screening factor for autosomal-recessive isolated ectopia lentis [18, 19], ADAMTSL4 has been mainly reported to participate in the microfibril formation and function [14, 20]. Recently, we revealed that ADAMTSL4 was highly expressed in the glioma stem-like cells and made a great contribution in the signature predicting the survival of GBM (WHO grade IV) [21]. Here, we probed into the biological processes influenced by ADAMTSL4 to further study the characteristics of ADAMTSL4 in GBM (WHO grade IV) and excavate its prognostic predicting potential in GBM (WHO grade IV).

In this study, we first tested the expression of ADAMTSL4 between normal brain tissue, lower-grade gliomas (LGG), and GBM (WHO grade IV). Then, we tried to explore the relationship between existing biomarkers of GBM (WHO grade IV), TCGA molecular subtypes, and ADAMTSL4. In addition, we analyzed the ADAMTSL4 correlated genes by DAVID website. Biomedical analyses revealed that ADAMTSL4 is strongly associated with immune-related processes. Then, we studied the relationship between immune status and ADAMTSL4 by testing the correlation between immune checkpoints and ADAMTSL4. Furthermore, we tested the infiltrated immune cells and tumor microenvironment applying the ESTIMATE, TIMER, and CIBERSORT. Finally, we found that high expression of ADAMTSL4 is an independent indicator of poor prognosis in GBM (WHO grade IV) patients.

## 2. Materials and Methods

### 2.1. Patients and Data Collection

In this study, we collected 591 pGBM cases from four main databases: CGGA (China), TCGA (the United States), REMBRANDT (the United States), and GSE16011 (the Netherlands).

The CGGA cohort included 88 patients including molecular and clinical information obtained from the CGGA database (http://www.cgga.org.cn) [22]. Two neuropathologists were enrolled to diagnose each patient, based on the 2007 WHO classification guidelines. Tumor samples were collected from newly resected tissue, and only those with more than 80% tumor cells were selected. Transcriptome sequencing data of GBM (WHO grade IV) samples were generated on the Illumina Hiseq 2000 platform. Overall survival (OS) was calculated from the diagnosing date to the death date or latest follow-up date. The patient characteristics are described in Table 1. This study was approved by the Beijing Tiantan Hospital institutional review board (IRB), and handwriting informed consent was acquired from each patient.

The TCGA RNA-seq data including 166 pGBM patients was downloaded from TCGA official website (http://cancergenome.nih.gov/). Both the GSE16011 (151 cases included) [23] and REMBRANDT (186 cases included) [24] were acquired from GEO website (https://www.ncbi.nlm.nih.gov/geo/).

In addition, the GEPIA software (a website analyzing the RNA sequencing expression data from the TCGA and GTEx projects) [25] was used to online analyze the expression difference between GBM (WHO grade IV) and normal brain tissues from its official website (http://gepia.cancer-pku.cn/detail.php).

### 2.2. Detection of GBM (WHO Grade IV) Biomarkers

In the CGGA cohort, both IDH1/2 mutation status and MGMT methylation status were detected by pyrosequencing, which is commonly used in clinical practice, following procedures described in our previous study [26, 27]. For the TCGA cohort, all molecular information was directly downloaded from the TCGA official website.

### 2.3. Immune-Related Analysis

ADAMTSL4-related gene sets were submitted to the DAVID website (https://david.ncifcrf.gov/summary.jsp) to perform GO analysis and investigate the relevant biological processes. Pearson's correlation analysis was used to evaluate the relationship between ADAMTSL4 and immune checkpoints. An R package—ESTIMATE (https://bioinformatics.mdanderson.org/main/ESTIMATE:Overview)—was used to demonstrate the presence and infer the fraction of stromal and immune cells in tumor samples. Tumor purity was calculated according to the formula described in Yoshihara and colleagues. We employed CIBERSORT to estimate the proportion of immune cell types in a mixed cell population online (https://cibersort.stanford.edu/). TIMER was also adopted to explore the correlation between gene expression and abundance of immune infiltrates (https://cistrome.shinyapps.io/timer/).

### 2.4. Statistical Analysis

The expression difference between subgroups was performed by unpaired Student's *t*-test. The prognosis analysis in four tremendous databases was evaluated by Kaplan-Meier curve. All statistical analyses were performed with the R (https://www.r-project.org/, v3.4.3), SPSS 16.0 (SPSS Inc., Chicago, IL), and GraphPad Prism 7 (GraphPad Software Inc., La Jolla, CA). The *p* value of 0.05 was taken as the significant threshold in all tests.

## 3. Result

### 3.1. ADAMTSL4 Was Enriched in GBM WHO Grade IV

**Figure 1:**
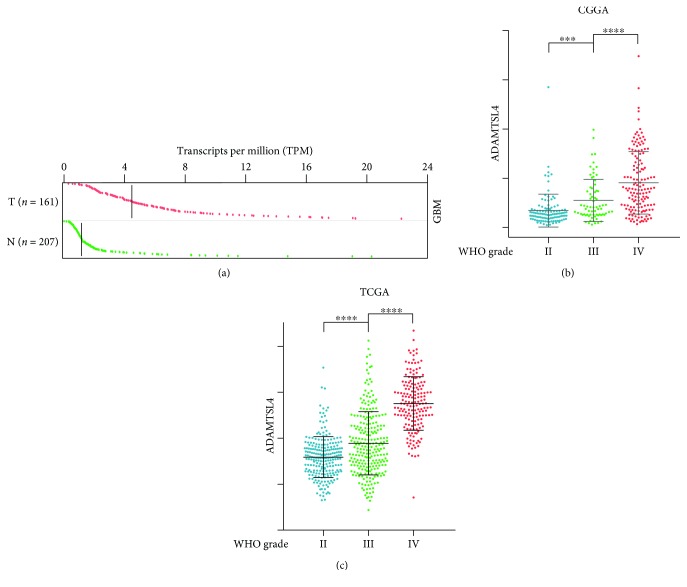
ADAMTSL4 was enriched in GBM (WHO grade IV). (a) ADAMTSL4 was significantly higher expressed in GBM (WHO grade IV) to normal brain tissue and analyzed by GEPIA website (T: GBM WHO grade IV tumor; N: normal brain tissue). (b, c) ADAMTSL4 expression is significantly upregulated along with the WHO grade in the CGGA database (b) and TCGA dataset (c). 109 WHO grade II, 72 WHO grade III, and 144 WHO grade IV patients in the CGGA database and 223 WHO grade II, 245 WHO grade III, and 168 WHO grade IV patients in the TCGA database. ∗∗∗ and ∗∗∗∗ indicate *p* < 0.001 and *p* < 0.0001, respectively.

To inspect the role of ADAMTSL4 in glioma, the expression level was first analyzed between normal brain tissue and GBM (WHO grade IV) using GEPIA website. We found that ADAMTSL4 expressed significantly higher in GBM (WHO grade IV), compared to normal brain tissue (*p*<0.05, Figure 1(a)), indicating ADAMTSL4 plays an important role in glioma oncogenesis. Due to the prominent heterogeneity across different glioma grades, the ADAMTSL4 expression from both CGGA and TCGA database was analyzed according to the WHO grade. In the CGGA RNA-seq database, ADAMTSL4 was significantly higher expressed in GBM (WHO grade IV) compared to lower-grade gliomas (LGG, including grade II and grade III gliomas) (*p* < 0.001, Figure 1(b)). This was also validated in the TCGA cohort (*p* < 0.0001, Figure 1(c)), which indicated that higher ADAMTSL4 expression was enriched in GBM and may play a critical role in the malignant progression of glioma.

### 3.2. ADAMTSL4 Was Correlated with IDH1/2 Mutation Status and MGMT Methylation Status in GBM WHO Grade IV

**Figure 2:**
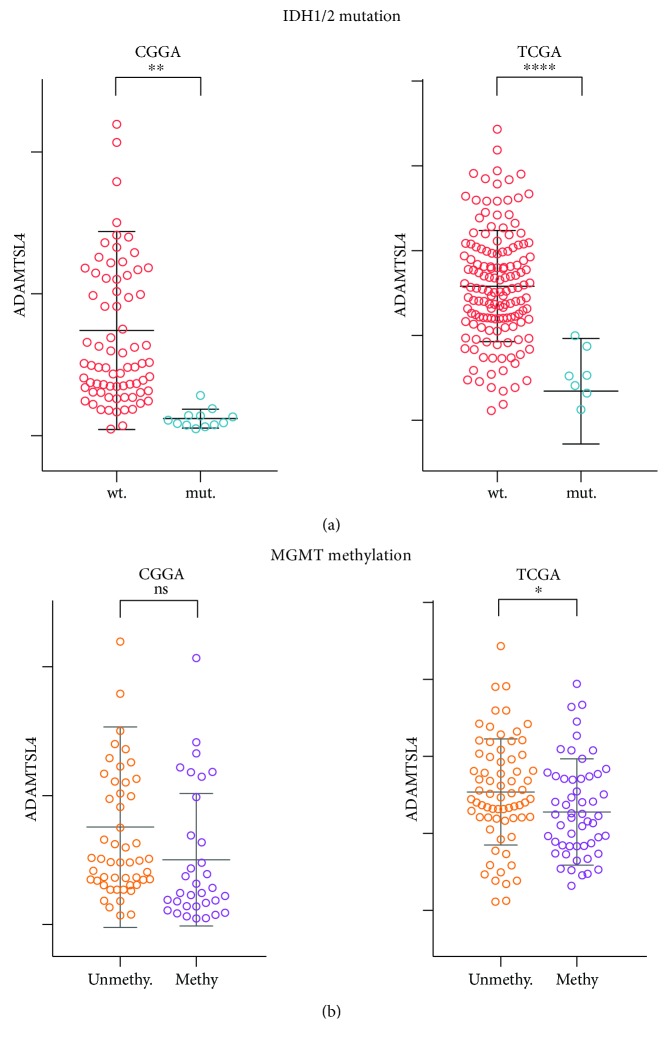
ADAMTSL4 was correlated with existing biomarkers in pGBM (WHO grade IV). ADAMTSL4 was significantly increased in IDH1/2 wild-type pGBMs both in the CGGA and TCGA database (a). ADAMTSL4 was significantly increased in MGMT unmethylated group in the TCGA database, but no significance was tested in the CGGA database (*p* = 0.1033) (b). ns, ∗, ∗∗, and ∗∗∗∗ indicate *p* > 0.05, *p* < 0.05, *p* < 0.01, and *p* < 0.0001, respectively.

The IDH1/2 mutation status and MGMT methylation status are playing vital role in the prognosis and chemotherapy of GBM (WHO grade IV) and showing great difference between individuals [28, 29]. In consideration of the prominent molecular heterogeneity of GBM (WHO grade IV), we further tested the ADAMTSL4 expression in GBMs with different IDH1/2 mutation status and MGMT methylation status, respectively. As a result, patients with IDH wild-type had higher ADAMTSL4 expression than those with IDH1/2 mutant in both CGGA (*p* < 0.01) and TCGA cohort (*p* < 0.0001, Figure 2(a)). When considering the MGMT methylation status in the CGGA cohort, we found that the methylated group has a trend toward lower ADAMTSL4 expression, comparing to the unmethylated group (*p* = 0.1033, Figure 2(b)). However, the ADAMTSL4 expression was dramatically decreased in the MGMT methylated group compared to the unmethylated group in the TCGA database (*p* < 0.05, Figure 2(b)). All of these findings indicated that ADAMTSL4 is closely associated with IDH1/2 mutation status and MGMT methylation status in GBM (WHO grade IV). Moreover, ADAMTSL4 was found specifically expressed in GSC cells in our earlier study [21].

### 3.3. ADAMTSL4 Was a Potential Marker for Malignant Subtypes in GBM (WHO Grade IV)

**Figure 3:**
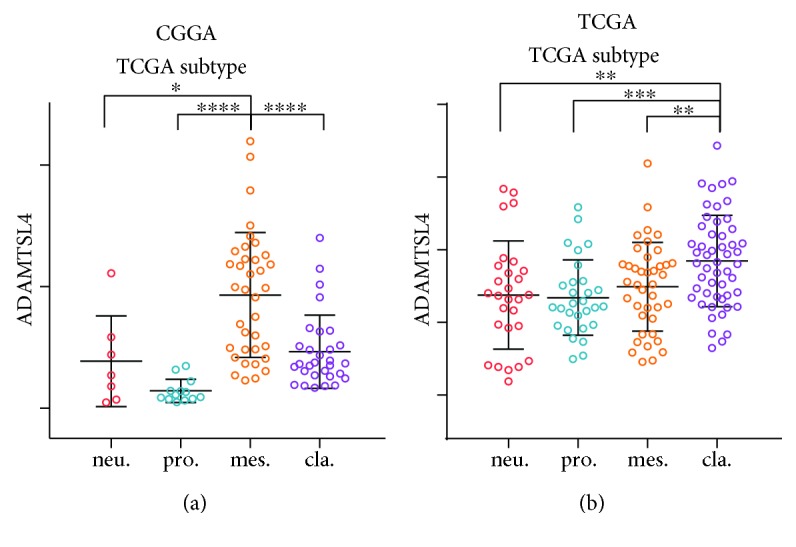
ADAMTSL4 was a potential marker for malignant subtypes in pGBM (WHO grade IV). ADAMTSL4 was highly expressed in mesenchymal subtype in the CGGA database (a) and in classical subtype in the TCGA database (b). ∗, ∗∗, ∗∗∗, and ∗∗∗∗ indicate *p* < 0.05, *p* < 0.01, *p* < 0.001, and *p* < 0.0001, respectively.

To explore the molecular expression pattern of ADAMTSL4 in GBM (WHO grade IV), we tested the distribution of ADAMTSL4 expression in different molecular subtypes defined by TCGA network [30]. ADAMTSL4 was significantly upregulated in the mesenchymal subtype than other subtypes in the CGGA dataset (Figure 3(a)). While in the TCGA database, the classical subtype was showed with the highest expression of ADAMTSL4 with great significance compared to other subtypes (Figure 3(b)). To our knowledge, both mesenchymal and classical subtypes were more malignant with poorer prognosis for GBM (WHO grade IV) patients. Therefore, we inferred that ADAMTSL4 might play the oncogenic role and result in different molecular patterns in GBM (WHO grade IV). To validate the hypothesis, biological function analyses were subsequently performed.

### 3.4. ADAMTSL4 Was Strongly Associated with Immune-Related Processes in GBM (WHO Grade IV)

**Figure 4:**
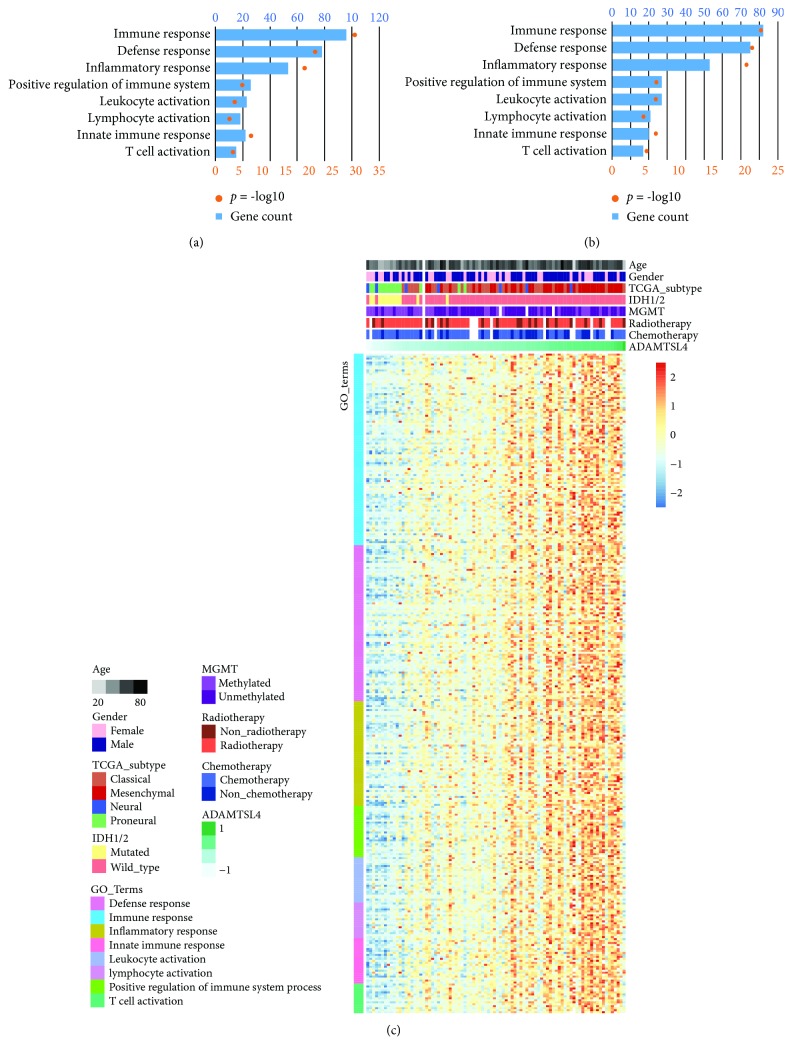
ADAMTSL4 was closely related to immune processes in pGBM (WHO grade IV). Immune process-related biological functions were enriched by ADAMTSL4 positively correlated genes both in the CGGA and TCGA databases (a, b). Most immune process-related genes were significantly positively correlated with ADAMTSL4 expression in the CGGA databases (c).

To explore the ADAMTSL4-related biological functions in GBM (WHO grade IV), we first identified the genes highly correlated with ADAMTSL4 (Pearson *R* > 0.4, *p* < 0.05) by Pearson's correlation analysis. Totally, 777 and 586 genes were identified in the CGGA and TCGA datasets, respectively. Subsequently, we investigated the two gene sets in Gene Ontology analysis using the DAVID online tool. As the result, we found that the genes positively correlated with ADAMTSL4 were enriched in biological functions (Figures 4(a) and 4(b)), including immune response, defense response, and other immune-related processes. The heat map of genes in each biological process showed obviously positive correlation to the expression of ADAMTSL4 in both two datasets (Figure 4(c), Figure S1). These findings suggested that ADAMTSL4 could be used in predicting the immune-related biological processes in GBM (WHO grade IV).

### 3.5. ADAMTSL4 Was Correlated to Immune Checkpoints in GBM (WHO Grade IV)

**Figure 5:**
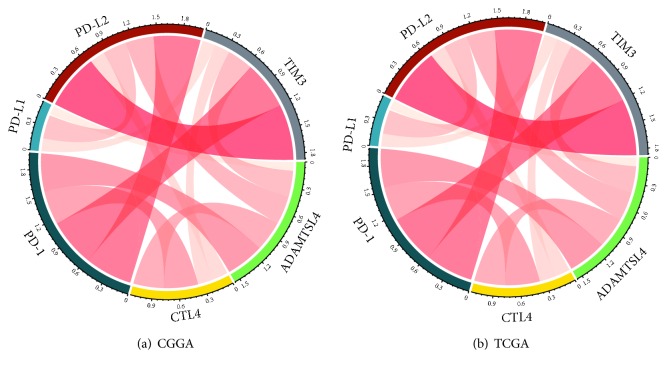
ADAMTSL4 was synergistic with immune checkpoints in tumor-induced immune response. Strong correlation between immune checkpoints and ADAMTSL4 expression was found both in the CGGA and TCGA databases (a, b).

As shown above, ADAMTSL4-related genes were found to be strongly associated with immune-related biological processes in GBM (WHO grade IV). Therefore, we further investigated the relationship between known immune checkpoint genes, including PD-1, PD-L1, PD-L2, TIM3, CTLA4, and ADAMTSL4. Coexpression analysis was performed among these six genes in both the CGGA and TCGA datasets. The result indicated that the genes were shown as high correlation with each other, especially between PD1 and ADAMTSL4 in the CGGA database (Figure 5(a)). Similar coexpressed pattern was validated in the TCGA database (Figure 5(b)).

### 3.6. ADAMTSL4 Revealed More Infiltrated Immune Cells but No Change in the Proportion of Immune Cells

**Figure 6:**
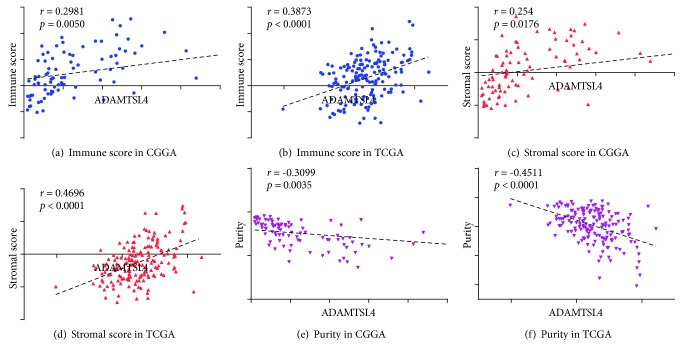
ADAMTSL4 revealed more infiltrated immune cells but no change in the proportion of immune cells. Scatterplot showed significant positive correlation between immune score and ADAMTSL4 expression in two databases (a, b). Significant positive correlation was also found between stromal score and ADAMTSL4 expression in two databases (c, d). Calculated tumor purity was negatively correlated with ADAMTSL4 expression (e, f).

The altered immune response could induce immune cell infiltration and complicate the tumor microenvironment. To infer the fraction of immune and stromal cells of each case, the R package - ESTIMATE - was applied in the CGGA database for immune and stromal score. The results showed that both the immune and stromal scores were positively correlated with the ADAMTSL4 expression (Figures 6(a) and 6(c)). In addition, the ADAMTSL4 expression was shown with lower tumor purity in the CGGA database (Figure 6(e)). All the results described above were validated in the TCGA database (Figures 6(b), 6(d), and 6(f)). We also estimated the abundance of infiltrated immune cells by CIBERSORT. Neither in CGGA nor in TCGA database, there was no significant correlation between neither kinds of immune cells with ADAMTSL4 (Figure S2). Consistent result was gained from TIMER, another algorithm analyzing tumor-infiltrating immune cells within TCGA database (Figure S3) and further verified the observation.

### 3.7. ADAMTSL4 Predicted Poorer Survival in GBM (WHO Grade IV)

**Figure 7:**
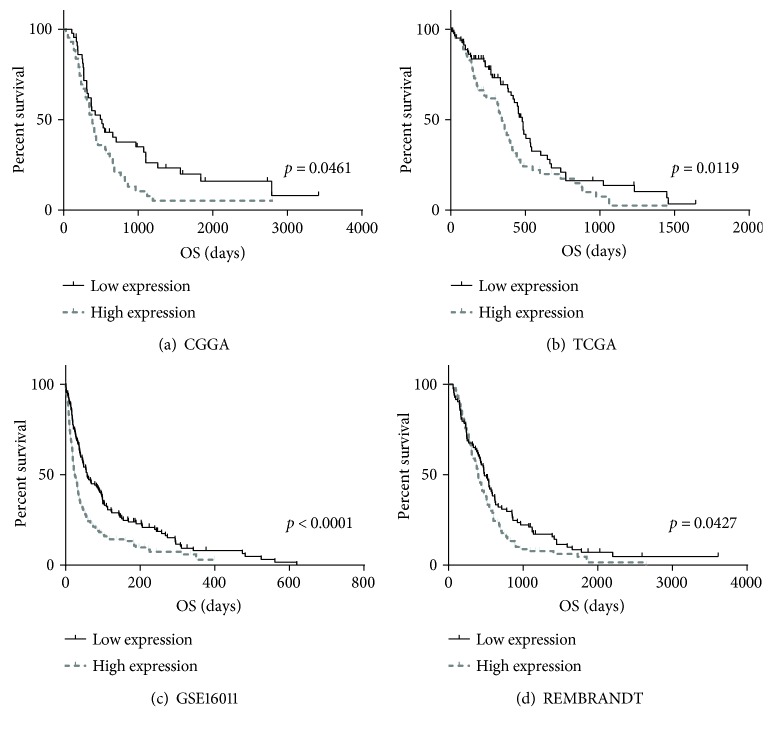
ADAMTSL4 predicted worse survival in GBM (WHO grade IV). The overall survival of high- and low-ADAMTSL4 expression in the CGGA (a), TCGA (b), REMBRANDT (c), and GSE16011 (d) databases is quite different.

**Figure 8:**
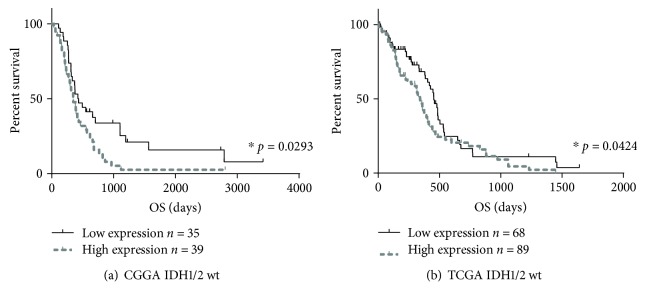
ADAMTSL4 predicted worse survival in IDH1/2 wild-type GBM WHO grade IV. The overall survival of high- and low-ADAMTSL4 expression in the CGGA (a) and TCGA (b) database is quite different. IDH1/2 wt: IDH1/2 wild-type.

In consideration of the strong relationship between ADAMTSL4 and immune status, we further analyzed the prognostic value of ADAMTSL4 in four databases. Patients with higher expressed ADAMTSL4 showed significant shorter overall survival (OS) than the counterparts in all four databases (*p* < 0.05 in CGGA, *p* < 0.05 in TCGA, *p* < 0.0001 in GSE16011, and *p* < 0.05 in REMBRANDT, Figures 7(a) and 7(d)). Considering the WHO 2016 classification [24, 28], we further analyzed the predictive effect in subgroups with wild-type IDH1/2 status in the CGGA and TCGA databases. In wild-type IDH1/2 subgroup, ADAMTSL4 also showed great predictive effect (*p* < 0.05 in both CGGA and TCGA, Figures 8(a) and 8(b)). We did not analyze the survival of patients with IDH-mutant GBM because of the limited patient numbers (12 in CGGA and 8 in TCGA database). Furthermore, uni- and multivariate Cox regressions were performed in the CGGA database, verifying the independence of the clinical prognostic significance of ADAMTSL4 in GBM (WHO grade IV). The expression of ADAMTSL4 showed significance in both uni- and multivariate Cox regressions (*p* < 0.01 in univariate Cox regression and *p* < 0.05 in multivariate Cox regression, Table 2). All these findings showed that ADAMTSL4 could be an independent biomarker to predict poor prognosis in primary GBM (WHO grade IV) by revealing more complicated immune status and tumor microenvironment.

## 4. Discussion

Nowadays, although comprehensive treatment of GBM (WHO grade IV) has been carried out to each patient, the prognosis is still poor [1, 2, 31]. With the development of immunotherapy, more and more attention is attracted to the heterogeneous immune status of GBM (WHO grade IV). Although several biomarkers from local tumor tissue have been identified nowadays, the role of circulating biomarker remains unclear.

In this study, we found that the expression of ADAMTSL4, as a secreted glycoprotein, significantly increased in GBM (WHO grade IV) compared to LGG, indicating its oncogenic role. We also found the strong correlation to IDH mutation and MGMT methylation, suggesting ADAMTSL4 to indicate the malignant molecular characteristics of GBM (WHO grade IV). Upregulated expression of ADAMTSL4 in mesenchymal and classical subtypes, revealed the infiltration of immune and stromal cells. All of these were further verified by the analyses of estimated immune score and stromal score. In addition, we also revealed that ADAMTSL4 is strongly correlated to the immune-related biological processes in GBM (WHO grade IV), including “immune response,” which means any immune system process that functions in the calibrated response of an organism to a potential internal or invasive threat. The coexpression analysis also showed great correlation between ADAMTSL4 and immune checkpoints. All of these indicated that ADAMTSL4 is a potential circulating biomarker not only for the prognosis but also for the immune status of GBM (WHO grade IV), which may direct the immunotherapy of GBM (WHO grade IV).

Immune checkpoints are the regulators of immune system, which are crucial for preventing the immune system from attacking cells indiscriminately [32]. With the development of immunotherapy, more than 20 immunologic molecules, especially drugs against CTLA-4 and PD-1/PD-L1 checkpoint pathways, were designed, shining the gloomy prognosis of malignant tumors [33, 34]. Here, we found strong correlation between ADAMTSL4 and immune checkpoints, especially between PD1 and ADAMTAL4, implying that ADAMTAL4 could be used to predict the status of PD1/PD-L1. All the results indicated the promising value of ADAMTSL4 in accessing compromised immune status in GBM (WHO grade IV). Thus, ADAMTSL4 could be used as a potential marker predicting the response of GBM (WHO grade IV) to immune therapy targeting immune checkpoints.

The tumor microenvironment does not only include tumor cells but also infiltrated immune cells, fibroblasts, and stromal cells comprising the tumor volume [35]. Several studies suggested that lower tumor purity is frequently linked to aggressive characteristics [26, 36]. In this study, we found that both the immune and stromal scores were positively correlated to the ADAMTSL4 expression, suggesting that ADAMTSL4 could reflect the increased fraction of immune and stromal cells. Meanwhile, we did not find ADAMTSL4 to be significantly correlated to the changes of any specific immune cell. The results indicated that ADAMTSL4 could effectively reflect the general changes of tumor microenvironment, but not linking to any specific immune cell activity. Recently, we revealed that ADAMTSL4 was upregulated in the glioma stem-like cell lines compared with conventional glioma cell lines [21]. Here, we uncovered the correlation between ADAMTSL4 and general changes of tumor microenvironment in pGBM. These indicated that potential links may exist between glioma stem-like cells and changed tumor microenvironment.

ADAMTSL4 mutation was reported to participate in the formation of ectopia lentis [14, 20]. While in GBM, we found that the increased ADAMTSL4 expression but not mutation is an independent prognostic indicator, for we had not observed the mutation of ADAMTSL4 in GBMs from the TCGA dataset (data not shown). So, whether the expression level of ADAMTSL4 could also be a marker in extopia lentis still needs investigation in future study.

In summary, ADAMTSL4 mainly enriched in the wild-type IDH1/2, unmethylated MGMT, and malignant GBM molecular subtypes. Investigation of the involved biological characteristics revealed the consistency of ADAMTSL4 with immune response, induced infiltration of immune cells, and tumor microenvironment. Although sufficient bioinformatic profiling has revealed the robust predictive value of ADAMTSL4, it is still not valid enough. Further experimental research should be followed to verify the results in our research.

## 5. Conclusion

In conclusion, bioinformatic analyses revealed that the expression of ADAMTSL4 is regulated by multiple mechanisms and mainly involved in immune processes. Higher ADAMTSL4 expression implies worse prognosis of primary GBM (WHO grade IV) patients, correlated with more intensive immune response and complicated tumor microenvironment. As a secreted glycoprotein of molecular property, ADAMTSL4 may be a potential circulating biomarker for primary GBM (WHO grade IV) to direct immunotherapy in the future, deserving further experimental research.

## Figures and Tables

**Table 1:** Clinical and molecular characteristics of 88 patients in the CGGA cohort.

Total variables (*n*, percentage)	pGBM (*n* = 88)
Age	
Median (range)	49.6 (12–81)
Age ≥ 45	31 (35%)
Age < 45	56 (64%)
NA	1 (1%)
Gender	
Male	55 (63%)
Female	32 (36%)
NA	1 (1%)
IDH1/2 status	
Mutation	12 (14%)
Wild-type	75 (85%)
NA	1 (1%)
MGMT promoter status	
Methylated	33 (38%)
Unmethylated	51 (58%)
NA	4 (4%)
Radiotherapy	
Yes	58 (66%)
No	18 (20%)
NA	12 (14%)
Chemotherapy	
Yes	51 (58%)
No	24 (27%)
NA	13 (15%)
TCGA subtype	
Neural	7 (8%)
Proneural	13 (15%)
Classical	31 (35%)
Mesenchymal	36 (41%)
NA	1 (1%)

IDH1/2 = isocitrate dehydrogenase 1; MGMT = methylguanine methyltransferase.

**Table 2:** Uni- and multivariate Cox regression analysis of the risk score and clinical information for OS in CGGA.

Variable	Univariate Cox			Multivariate Cox		
	*p* value	HR	95% CI for HR	*p* value	HR	95% CI for HR
Age						
≥45 vs. <45	0.3486	1.2638	0.7746-2.0618			
Gender						
Male vs. female	0.2991	1.3016	0.7915-2.1404			
IDH1/2 status						
Mutation vs. wild-type	0.0465	0.4678	0.2214-0.9884	0.6466	0.7800	0.2697-2.2561
MGMT promoter status						
Methylated vs. unmethylated	0.0479	0.6062	0.3692-0.9954	0.0609	0.4085	0.2407-1.0325
Radiotherapy						
Yes vs. no	0.3621	0.7629	0.4263-1.3654			
Chemotherapy						
Yes vs. no	0.0440	0.5788	0.3400-0.9854	0.0976	0.5818	0.3065-1.1042
KPS score						
Increasing score	0.0057	0.9733	0.9548-0.9922	0.0020^∗^	0.9663^∗^	0.9456-0.9876^∗^
ADAMTSL4 expression						
Increasing expression	0.0061^∗^	1.2891^∗^	1.0753-1.5454^∗^	0.0289^∗^	1.3224^∗^	1.0293-1.6990^∗^

HR = hazard ratio; IDH1/2 = isocitrate dehydrogenase 1; MGMT = methylguanine methyltransferase; KPS = Karnofsky performance score. Factors with prognostic significance in univariate Cox regression analysis were included in further multivariate Cox analysis. ^∗^Significant.

## Data Availability

The data used to support the findings of this study are included within the article.

## Supplementary Materials

Supplementary Materials Figure S1: ADAMTSL4-related genes enriched in immune-related GO terms. Most immune process-related genes were significantly positively correlated with ADAMTSL4 expression in the TCGA databases. Figure S2: the composition of infiltrated immune cells was estimated by CIBERSORT. The fraction of different types of infiltrated immune cells was estimated by CIBERSORT in both two databases (A, B), while no correlation that was found between neither type of immune cells and ADAMTSL4 expression was tested by Pearson's correlation analysis. Figure S3: correlation between gene expression and abundance of immune infiltrates in TCGA. Correlation between different types of infiltrated immune cells and ADAMTSL4 expression was estimated by TIMER.
