# Supplementary material for: ADAMTSL4, a Secreted Glycoprotein, Is a Novel Immune-Related Biomarker for Primary Glioblastoma Multiforme
Source: Dis Markers. 2019 Jan 8;2019:1802620. doi: 10.1155/2019/1802620 (PMC6341252; doi:10.1155/2019/1802620)

# Supplementary Materials

Fig. S1 ADAMTSL4 related genes enriched in immune related GO terms. Most immune process related genes were significantly positively correlated with ADAMTSL4 expression in the TCGA databases.

Fig. S1 ADAMTSL4 related genes enriched in immune related GO terms

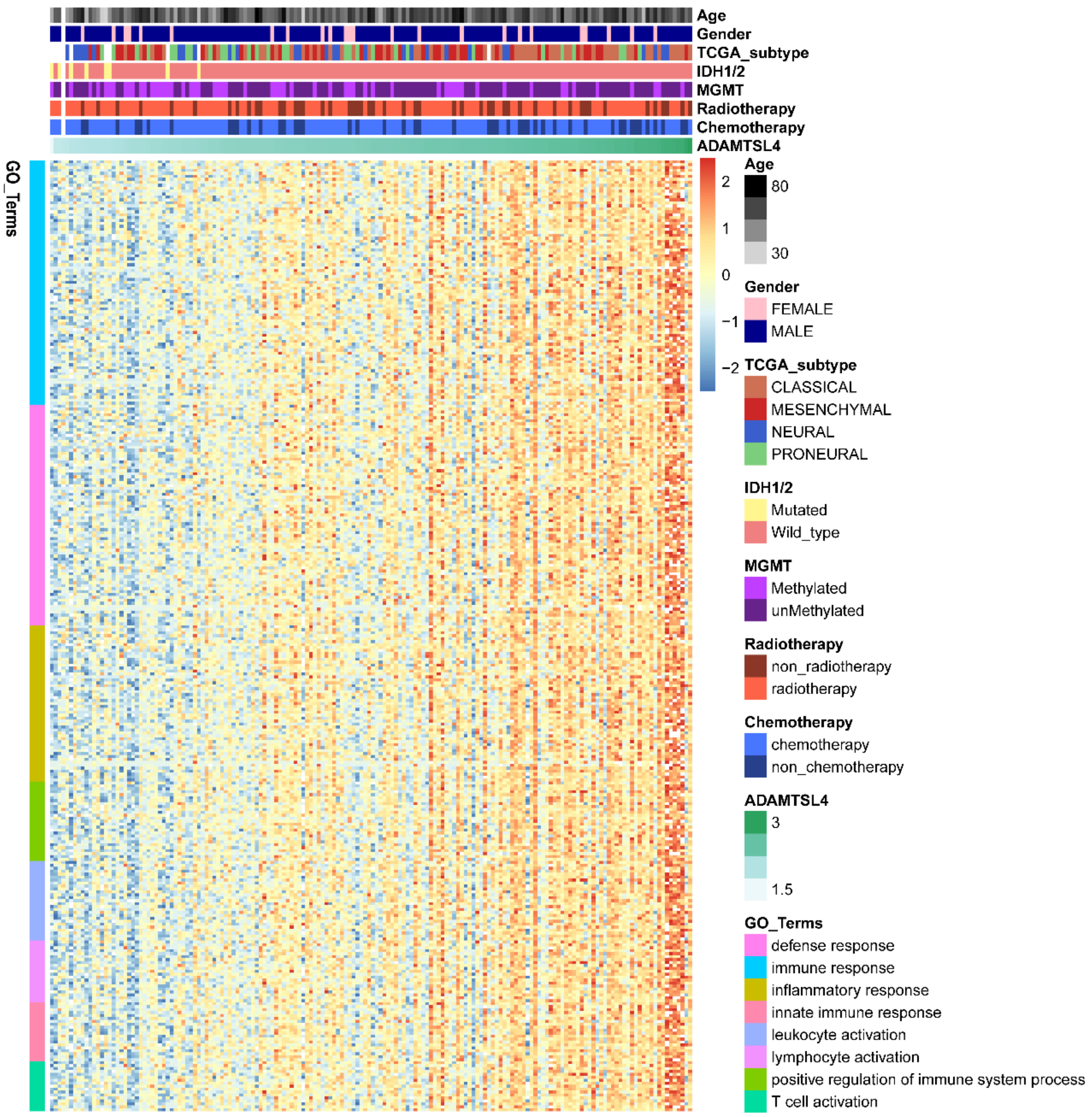

Fig. S2 The composition of infiltrated immune cells was estimated by CIBERSORT. The fraction of different types of infiltrated immune cells was estimated by CIBERSORT in both two databases (A, B). While no correlation was found between neither type of immune cells and ADAMTSL4 expression was tested by Pearson correlation analysis.

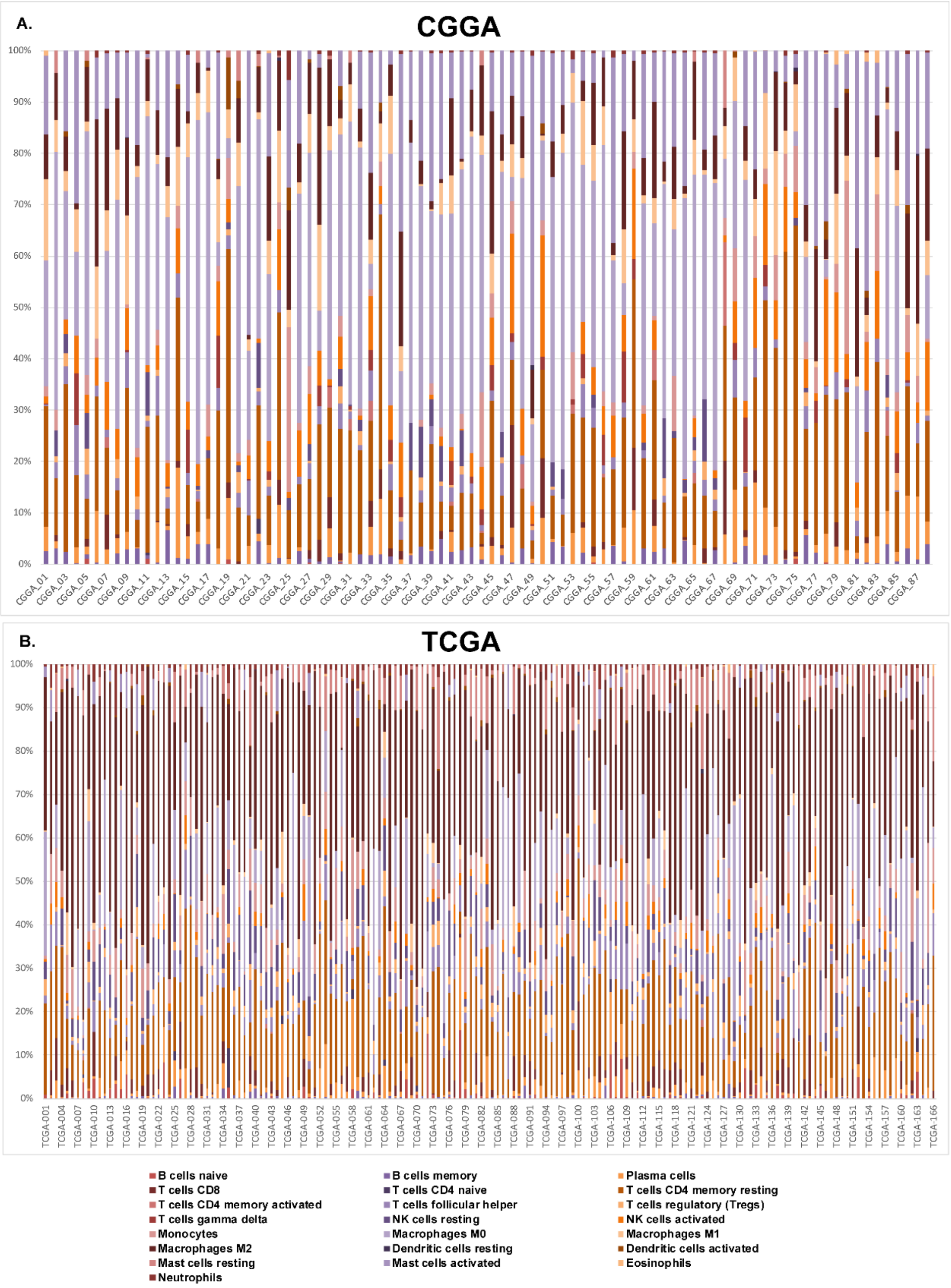

Fig. S3 Correlation between gene expression and abundance of immune infiltrates in TCGA. Correlation between different types of infiltrated immune cells and ADAMTSL4 expression was estimated by TIMER.

**Fig. S3 Correlation between gene expression and abundance of immune infiltrates in TCGA**

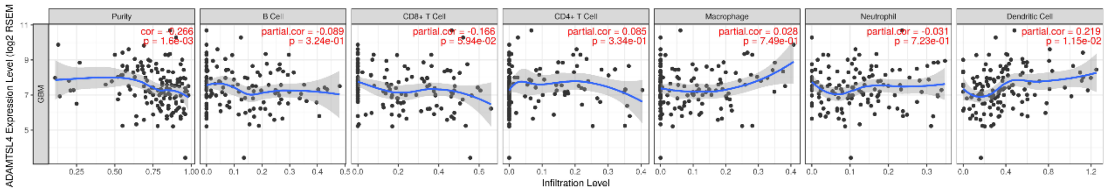

Supplement: Supplementary Materials — Figure S1: ADAMTSL4-related genes enriched in immune-related GO terms. Most immune process-related genes were significantly positively correlated with ADAMTSL4 expression in the TCGA databases. Figure S2: the composition of infiltrated immune cells was estimated by CIBERSORT. The fraction of different types of infiltrated immune cells was estimated by CIBERSORT in both two databases (A, B), while no correlation that was found between neither type of immune cells and ADAMTSL4 expression was tested by Pearson's correlation analysis. Figure S3: correlation between gene expression and abundance of immune infiltrates in TCGA. Correlation between different types of infiltrated immune cells and ADAMTSL4 expression was estimated by TIMER. [file 1802620.f1.pdf]
